# Dental anxiety, cardiovascular changes and patient preconceptions toward implants and root canal treatments: An observational study

**DOI:** 10.4317/jced.59281

**Published:** 2022-10-01

**Authors:** Carlota de España, Juan-Gonzalo Olivieri, Jordi Ortega-Martinez, Sergio Morelló, Miguel Roig-Cayón, Fernando Durán-Sindreu

**Affiliations:** 1 Department of Endodontics, Universitat Internacional de Catalunya, Barcelona, Spain; 2 Department of Restorative Dentistry, Universitat Internacional de Catalunya, Barcelona, Spain

## Abstract

**Background:**

There is little information about dental anxiety and the patient’s vital signs during dental procedures. This study evaluates and compare patient anxiety levels and cardiovascular changes before and during root canal treatment (RCT) and single-tooth implant procedures.

**Material and Methods:**

Preoperative data and pre-treatment considerations were recorded. HR and SpO2 were monitored during treatment procedures at five points. Data were analyzed accordingly using Mann Whitney or X2 tests. 80 patients met the inclusion criteria.

**Results:**

Anxiety and fear scores were strongly correlated (*p*< 0.001). Both treatments resulted in low levels of dental anxiety and fear. Patients with a prior dental bad experience presented higher anxiety scores in the implant treatment group (*p*< 0.05). Implant treatment was considered a more time-consuming and more painful procedure than root canal treatment (*p*< 0.05). No significant relation was found between the level of anxiety with HR and SpO2.

**Conclusions:**

No significant relation was found between the level of dental anxiety with HR and SpO2. Single-implant treatment was pre-considered to be a more time-consuming and more painful procedure when compared with a RCT. HR was higher at the initial stages and decreased as both procedures finished.

** Key words:**Dental anxiety, endodontics, dental implants, oximetry, heart rate.

## Introduction

The etiology of dental anxiety includes the social and psychological aspects of human cultural identity (1). Anxiety and fear are usually analyzed together, however, there are different reactions; fear is a concrete or activated response to a situation or object, whereas anxiety is the patient’s emotional state before confronting the feared situation or object (2).

The vast majority of the patients express their fear and stress during dental procedures (3). Levels reported in the literature were high and decreased with time (4). Anxiety has been identified as a potential barrier to accessing dental care, which is the primary reason for delaying or canceling appointments resulting in an increased risk of deterioration of the quality of oral health (5).

Moreover, high dental fear and anxiety levels have been reported in several recent studies to be around 5.5 - 15% of the population analyzed in several countries worldwide (6). Manifestations of this anxiety in patients during treatment include shortness of breath, tachycardia, hypertension, and hyperventilation among others (3).

Root canal treatment is a standard procedure in the daily clinical practice that patients still perceive with high levels of anxiety (7). Dental implant placement is one of the treatment choices to deal with tooth loss, with a high level of satisfaction but reportedly high patient anxiety (6). Pretreatment anxiety and the subsequent stress response may result in an increased pain sensation (8). Moreover, anxiety can make patients uncooperative and potentially arduous to manage (9). Thus, assessing and controlling anxiety levels before and during treatment can be crucial in ensuring adequate procedures achieving a favorable patient and dentist satisfaction.

There are no studies that evaluate cardiovascular changes and patient preconceptions during dental treatments or compare RCT and implant procedures in the aforementioned terms. Besides, to reduce patient anxiety during the treatment procedure, it is vital to know what treatments result in higher dental anxiety levels. Still, it has not been addressed if there is any difference between them or changes in the procedure’s different stages.

Thus, the present study aimed to evaluate and compare patient anxiety levels and changes in the patients’ heart rate (HR), and blood oxygen saturation (SpO2), before and during root canal treatment and single-tooth implant procedures. Secondary aims are to analyze possible relationships with patients’ fear, treatment decisions, and pretreatment considerations. The null hypothesis is that there are no differences in cardiovascular changes during RCT and implants.

## Material and Methods

The present is an observational cross-sectional study with a longitudinal design for heart rate and blood oxygenation levels evaluation at different points of the procedure. The guidelines were followed for observational studies of the reporting strengthening of observational studies in epidemiology. A total of 80 patients were included in the study.

Procedures were performed in private clinical practices with the accreditation of the competent authority to perform clinical studies and with the University Ethics Committee’s approved supervision from September 2019 to January 2020 (ENDECL201801E1). Two dentists conducted the procedure and registered patient data. One specialist in oral surgery with ten years of experience and one specialist in endodontics with eight years of experience. Only single dental implants and single root canal treatments were included.

Patients were identified according to the treatment considered as the treatment choice in each specific case. Once identified, criteria for inclusion were patients over 18 years old, willing to participate that fully understood and accepted to sign the informed consent, with no abnormal medical conditions, and root canal performed in one single visit. Exclusion criteria included the following: pregnant women, patients with psychiatric problems, patients with anxiolytic, antidepressant medication prescribed, non-controlled hypertensive patients, and patients that scored 19 or more on the modified dental anxiety scale (MDAS).

A two-group comparison was established according to the treatment performed, the root canal treatment group, and the implant group. Interactions of the following variables were also considered for analysis.

• Demographic variables: age and sex.

• Preoperative variables:

- MDAS

- Fear: from 0 to 10

- Anxiety: from 0 to 4 (general anxiety to dental treatments, anxiety the day before and the day of the treatment)

- Decision-making questionnaire including the influence of fear, price, treatment duration, aesthetics, and final decision-making

- Treatment considerations questionnaire including price, level of pain at the moment of performing the treatment and, treatment time

- HR and SpO2 (T1)

• Intraoperative variables:

- HR and SpO2 (T2: during anesthesia, T3: during root canal instrumentation or implant burs sequence, T4: during intraoral radiograph taking, and T5: at the end of the procedure)

- Full extension of the procedure in minutes.

All patients willing to participate were kindly asked to fill out the preoperative questionnaires in the waiting room. After completion, preoperative heart rate and oxygen saturation were measured while the patient was seated in the dental box chair with a portable fingertip pulsioximeter. The dental assistant recorded all the measurements before, during, and after the procedure. After, the dentists initiated the corresponding treatment procedure. All treatments were performed in a standardized manner. For both treatments anesthesia with articaine 40mg/ml (4%) epinephrine 1/100.000 was used.

Sample size calculation was performed based on MDAS results of a pilot study of 24 patients (11 RCT and 13 implant procedures) with a standard deviation of 2.58. We accepted an alpha and beta risk of 0.05 and 0.2, respectively, for a two-sided test of two groups of the same size ratio. Thirty-nine subjects resulted necessarily for each group, recognizing as statistically significant difference greater than or equal to two units, assuming a 10% of possible losses due to incomplete or incorrect data registering.

Data was transferred to an excel spreadsheet (Microsoft Corporation, Redmond, WA, USA). For data analysis, the metafor package version 2.0 of the R software (Free Software Foundation, Boston, MA, USA) was used. Frequency distributions of both groups were analyzed using X2 and T-Tests. According to data distribution, patient pretreatment questionnaire answers were analyzed using Mann Whitney or X2 tests regarding whether variables were categorical or continuous, to evaluate differences between groups. The level of statistical significance was set in 0.05.

## Results

A total of eighty patients included met inclusion/exclusion criteria, 40 men and 40 women. Patients’ mean age was 47.87 ± 15.48 years. Implant group included 15 men (mean age: 55.13 ± 12.2) and 25 women (mean age: 57.04 ± 12.4). RCT group included 22 men (mean age: 42.08 ± 14.09) and 18 women (mean age: 35.27 ± 11.44). RCT procedure was more time-consuming (50 ± 9 min.) when compared with the implant procedure (33.8 ± 8 min.) (*p* < 0.05).

Both MDAS and fear scores were strongly correlated (*p* < 0.001). Regarding anticipatory anxiety, no difference was found between treatment groups, age, sex, or tooth group (*p* > 0.05), except for a single implant in the anterior zone (*p* < 0.05). Patients with a prior dental bad experience presented higher MDAS scores in the implant treatment group (*p* < 0.05). No difference was found in patients with no previous bad experience (*p*< 0.05). Patients in both groups reported low levels of fear (*p* > 0.05). Female patients revealed higher levels of fear (*p* < 0.05), and males in the RCT group presented higher levels of fear when compared with males in the implant group (*p*< 0.05). If not having had a prior bad experiences, higher levels of fear were observed in the RCT group (*p* < 0.05).

The level of perceived anxiety regarding a visit to the dentist in general, the day before the visit, and the day of the procedure were 1.36 ± 1.1, 0.80 ± 1.0, and 1.46 ± 1.1, respectively. Values regarding the different groups are displayed in Table 1.

**Table 1:**
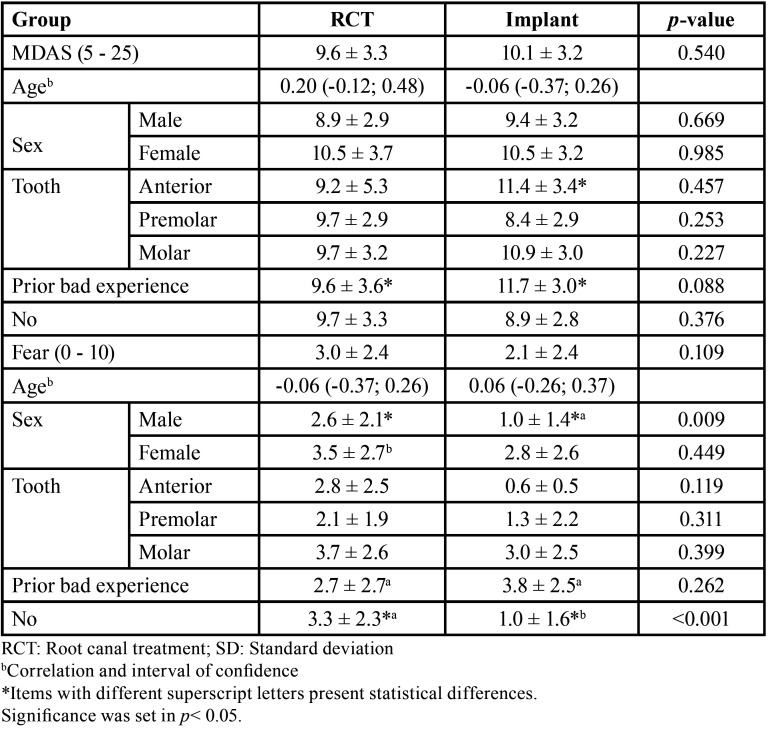
Anticipatory anxiety and fear in patients in both groups according to the treatment group and sex (Mean ± SD).

In terms of preconceived considerations regarding the treatment, the primary concern was that treatments were expensive. In general, both treatments’ time extension was not considered too extensive in time and was not perceived to cause high levels of pain. However, implant treatment was considered a more time-consuming and more painful procedure when compared with a root canal treatment (*p* < 0.05) (Table 2).

**Table 2:**
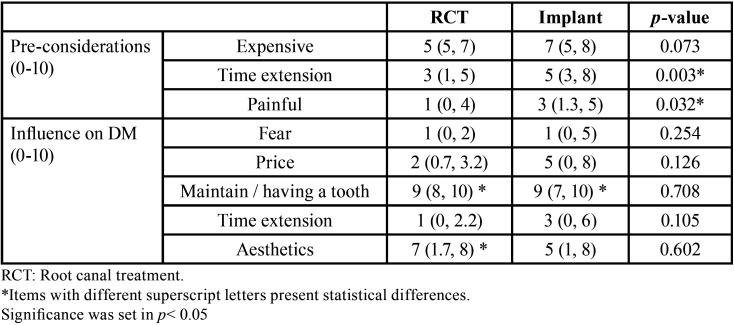
Questions regarding preconception towards the procedure and influence in treatment decision making (Median ± IR).

Regarding the decision or treatment choice, 75 % of the patients expressed that alternatives to the treatment selected were explained before the treatment choice (*p* < 0.05), with no differences between groups (*p* > 0.05). Also, 75 % of the treated patients perceived that the final treatment decision was made involving both the dentist and the patient (*p* < 0.05), with no differences among treatment groups (*p* > 0.05). When considering being treated, maintaining or having a tooth seems to be the most important factor, followed by aesthetics and price, in this order. Differences between groups are shown in Table 2.

Data of HR and SpO2 of the different treatment groups in the different time points are displayed in Table 3. No difference was found between groups in HR changes in the different time points (*p* > 0.05). No significant relation was found between the level of dental anxiety and fear with HR and SpO2 (*p* > 0.05).

**Table 3:**
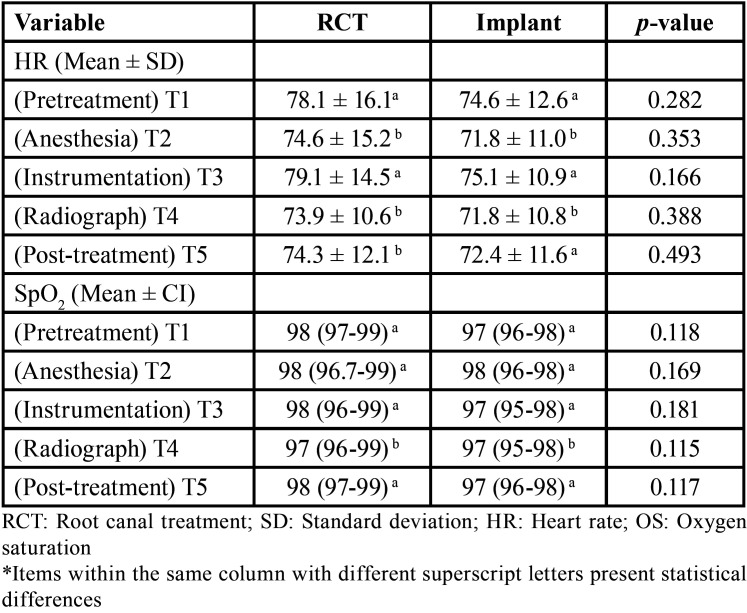
Heart rate (HT) and Oxygen saturation (SpO2) of patients in both groups in the different time points.

## Discussion

Dental anxiety and fear are concurrent problems worldwide and have been closely associated (6). Accordingly, in this study, both variables were strongly correlated in patients during both treatment procedures (*p* < 0.001). In our study, patients with prior dental bad experiences registered higher perceived dental anxiety levels in the implant group. Dental patients develop lower anxiety levels if they start with less painless treatments (9) and dental fear is lessened through regular dental visits (10).

Prevalence of dental fear and anxiety appears to be similar in different countries worldwide, and around 15-42 % of individuals have reported to experience high levels of dental fear (11). Our study mean of both groups anticipatory dental anxiety resulted in 9.8 ± 3.2, with no statistical differences between procedures. Other authors found similar results when facing dental treatments (12).

RCT is considered one of the treatments that result in higher dental anxiety levels compared with other therapies. In this study, patients in the RCT group reported a mean of 9.6 ± 3.3 MDAS, which is in accordance with data published in the literature (3). For patients in the implant procedure group, our results were higher to those reported by Kazancioglu *et al*. (13) but much lower than the 17.4 ± 3.5 reported by Camacho *et al*. (14). Differences could be related to the dental setting, where part University setting and part private practice were performed in their study.

When asked for their fear of the treatment, female patients reported having higher levels than male patients (*p* < 0.05) in both treatments. In general, women tend to be more anxious than men and show higher dental anxiety and fear (5). When evaluating anxiety towards RCT, Khan *et al*. (15) observed that in the majority of the studies, females presented higher scores. Higher medians were also found in this study, but with no statistical differences. Women experience higher stress and anxiety levels, partly related to a higher sensitivity to stressful and traumatic life experiences (16).

A portable fingertip pulse oximeter was used in the present study to evaluate patient changes in HR and SpO2 during RCT and implant procedures. According to our results, both treatments exhibit similar values of HR. In both groups, higher HR values were visible the moment before the start of the procedure and during the instrumentation or implant drilling sequence. This is in accordance with other studies where the HR was increased at the moment before a dental visit, and higher, when compared with values registered the day before (12). However, values were lower when compared with other procedures, such as a third molar tooth extraction (17). This can recognize that both RCT and implant procedures with actual devices performed by trained specialists are not very disturbing for the patient. Analyzing HR and SpO2 only at different specific moments during treatments could be a limitation of this study. Monitoring HR and SpO2 continuously during different dental treatments could help us to identify the exact moments where cardiovascular alteration occurs.

Anesthesia is considered one of the most fearful phases in dental treatments (18). In this study, 4% Articaine was used for both treatment procedures. It has been shown that it does not exert important effects on HR and oxygen saturation in healthy patients (19). HR can be altered by certain individual variables, including medication (9). Thus, patients taking any antidepressant medication were excluded.

Regarding SpO2 monitoring, no clinically relevant alterations occurred. Levels were maintained throughout both entire procedures. Some small differences were found between the two procedures, but can be only due to patient age. Constant SpO2 values have also been reported during more invasive dental procedures such as third molar extractions (20).

The main concerns among patients about implant procedures are the price, pain, and possible complications (21). Both treatments were considered expensive in our study, with no statistical differences (*p* > 0.05). However, the implant procedure was considered to be more painful and more time-consuming than RCT. This is a fact to highlight since it was not related to the real situation where the implant procedure was performed in statistically less time than RCT (*p* < 0.05). Patients consider dental implant procedures a high-quality treatment related to treatment costs.

Patients wish to be involved in healthcare decisions and take an active part in the decision-making process. Our results show that most patients (75%) declared that alternative treatment plans were also described before the final decision. Patients prefer collaborative decision in a professional-patient relationships (8).

## Conclusions

Within this study’s limitations, we can conclude that there were no differences in self-perception on dental anxiety and fear between patients undergoing a RCT and single-implant procedures. It can be stated that under normal conditions, RCT and single-implant procedures performed by trained specialists do not produce a significant alteration in HR and SpO2 values.
